# Chronic unpredictable stress induces anxiety-like behaviors in young zebrafish

**DOI:** 10.1038/s41598-020-67182-4

**Published:** 2020-06-25

**Authors:** Archana Golla, Henrik Østby, Florence Kermen

**Affiliations:** 1 Department of Biology, Faculty of Natural Sciences, Norwegian University of Science and Technology, 7491 Trondheim, Norway; 2 Kavli Institute for Systems Neuroscience and Centre for Neural Computation, Faculty of Medicine and Health Sciences, Norwegian University of Science and Technology, 7030 Trondheim, Norway

**Keywords:** Sensorimotor processing, Social behaviour, Stress and resilience

## Abstract

Exposure to stress during early life affects subsequent behaviors and increases the vulnerability to adult pathologies, a phenomenon that has been well documented in humans and rodents. In this study, we introduce a chronic unpredictable stress protocol adapted to young zebrafish, which is an increasingly popular vertebrate model in neuroscience research. We exposed zebrafish to a series of intermittent and unpredictable mild stressors from day 10 to 17 post-fertilization. The stressed fish showed a reduced exploration of a novel environment one day post-stress and an increased responsiveness to dark-light transition two days post-stress, indicative of heightened anxiety-related behaviors. The stress-induced decrease in exploration lasted for at least three days and returned to control levels within one week. Moreover, stressed fish were on average 8% smaller than their control siblings two days post-stress and returned to control levels within one week. All together, our results demonstrate that young zebrafish exposed to chronic unpredictable stress develop growth and behavioral alterations akin to those observed in rodent models.

## Introduction

The stress response is a combination of neural, endocrine and autonomic reactions that prepares an organism to cope with adverse events (stressors) that threaten its homeostasis. It results in an immediate release of catecholamines via the sympathetic nervous system followed by the rapid production of glucocorticoids via the hypothalamic-pituitary-adrenal (HPA) axis^1^. While the acute stress response is crucial for an animal’s adaptation to a changing environment, the repeated activation of the stress response within a short timeframe (chronic stress) contributes to a variety of adverse health effects including growth impairments, as well as mood and anxiety disorders^2,3^. In particular, animal and human studies have indicated that chronic stress exposure during early life increases the vulnerability to anxiety and depressive disorders via HPA axis dysregulation^4–8^. Since early-life adversity is an important risk factor for developing subsequent pathologies, understanding the neurobiological mechanisms underlying its adverse effects on the developing brain and behaviors is of utmost importance.

The zebrafish is rapidly becoming a popular model organism to study stress-induced changes in behaviors and neural circuits at early life stages^9–11^. Firstly, the main molecular and cellular components of the human stress response are conserved in zebrafish, including cortisol release^12–14^. Secondly, anxiety-related behavioral tests in zebrafish are relevant to understand anxiety disorders in mammals, including humans; for example, stressed zebrafish avoid the center of an openfield^15^, similar to center avoidance reported in humans with high anxiety sensitivity^16^. In addition, zebrafish larvae are optically transparent, which allows for rapid measurement of brain-wide neural activity at single-cell resolution during the first weeks of life^17,18^. Lastly, during this period, zebrafish already swim freely, feed externally and develop complex sensorimotor and social behaviors^19–21^, which are modulated by stress in adults^15,22,23^ and therefore enable to investigate the outcomes of chronic stress.

Leveraging these advantages, several recent studies have investigated how activation of the stress axis during early life affects zebrafish behavior. In post-hatch zebrafish, brief activation of the stress response by physiological stressors (acidic pH, hyperosmotic medium) or via optogenetic hypercortisolemia, resulted in transiently increased locomotion and stimulus responsiveness^13,24^ as well as temporary suppression of feeding behavior^25^. Upon longer exposure to inescapable mild electric shocks, young zebrafish initially attempted to escape before rapidly reducing their locomotor activity^26^, which is an example of a transition from active to passive coping strategies also found in other vertebrates^27^. Additionally, exposure to prolonged stressors such as 24 h exogenous cortisol or 9 h forced swimming, caused a heightened locomotor activity^28,29^ and a blunted stress-induced cortisol release^29^ in the following days. All together, these studies illustrate that the behavioral outcomes of early-life adversity vary depending on the nature, intensity, duration and timing of the stressors, and motivate further investigation of how early-life stress affects zebrafish behavior.

Despite a growing understanding of early-life stress in zebrafish, little is known about how chronic stress affects zebrafish during this sensitive developmental period. Therefore, our study aimed at establishing a chronic unpredictable stress protocol suitable for early life stages and at assessing how chronic stress exposure affects zebrafish development and anxiety-related behaviors.

## Results

### Repeated stressor exposure affects fish development

**Figure 1:**
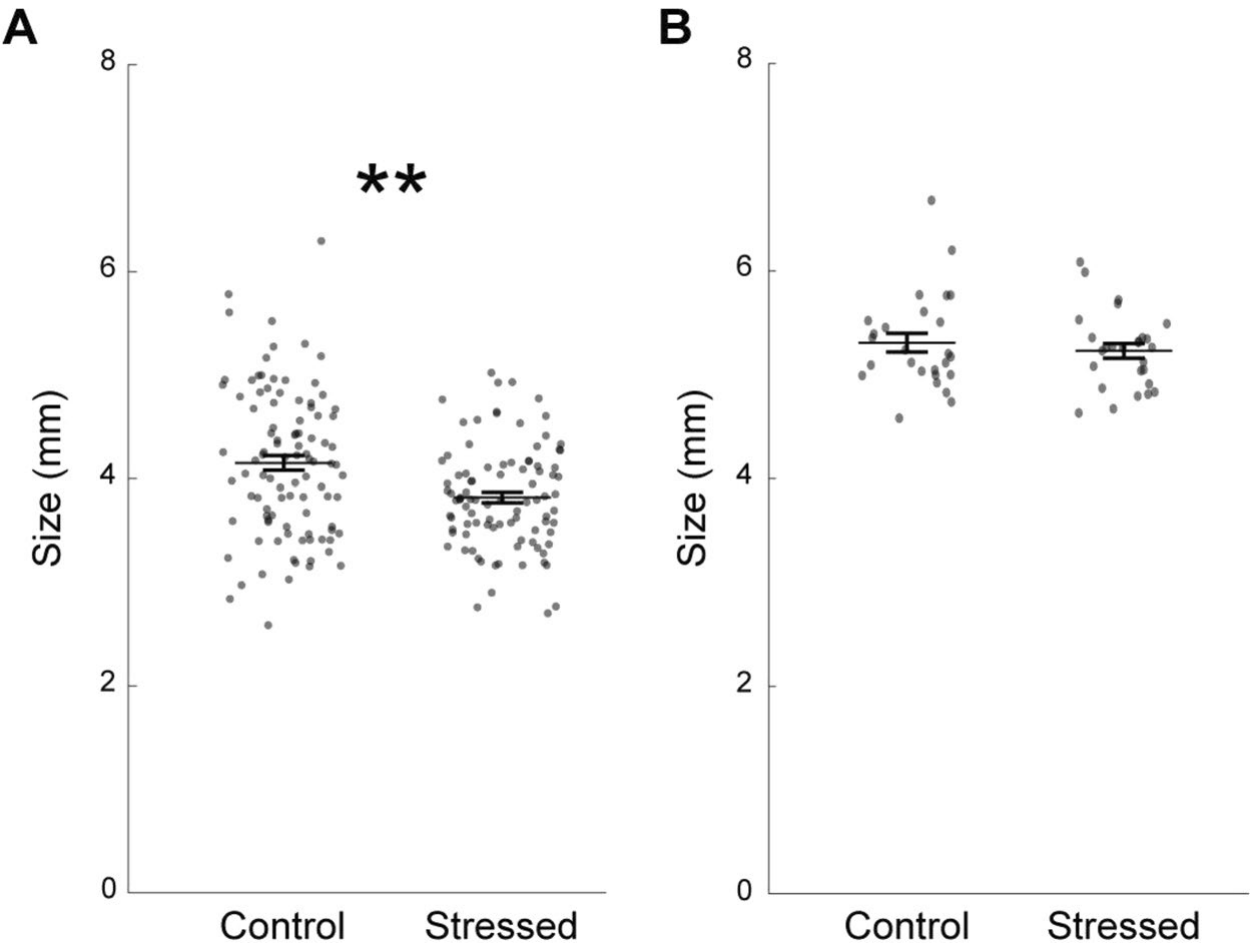
Stressed zebrafish are smaller two days post-CUS, but return to normal size within a week. (**A**) Fish size at 19 dpf (control: n = 99 fish; stressed: n = 94 fish). (**B**) Fish size at 25 dpf in a different set of fish (control: n = 27 fish; stressed: n = 26 fish). Each dot represents a fish. Data are represented as mean ± SEM. **p < 0.01, Kruskal-Wallis test.

We adapted a chronic unpredictable stress (CUS) paradigm previously used in adult zebrafish^22,30^, and exposed 10 days-post-fertilization (dpf) fish to intermittent stressors twice a day for eight consecutive days (see Methods). As previous studies have demonstrated that early life stress slows down development in fish^31,32^, we compared body length between groups of fish two days post-CUS and found that CUS-exposed fish were 8% smaller on average than their control siblings (Fig. 1A). No such difference was observed at eight days post-CUS (Fig. 1B). Therefore, CUS exposure temporarily slowed the young zebrafish development.

### Increased stimulus responsiveness in stressed zebrafish

**Figure 2:**
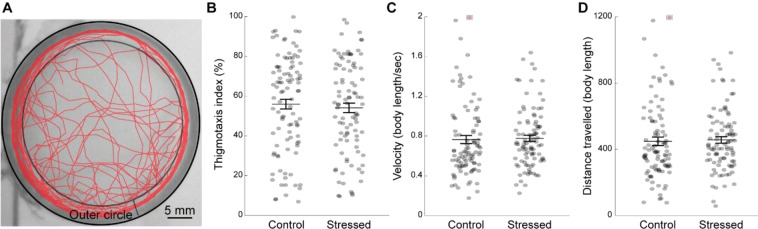
CUS exposure does not affect anxiety-like behaviors two days post-CUS in the open field test. (**A**) Representative swimming trajectory of a fish (in red) during the open field test lasting for ten minutes. The outer circle is indicated by two concentric black circles. (**B**) Thigmotaxis index that corresponds to the percentage of time spent in the outer circle. (**C**) Average swimming velocity in body length/second. The dot signaled by a red square was out of range (value = 2.78). (**D**) Total distance travelled for ten minutes in body length. The dot signaled by a red square was out of range (value = 1669). Each dot represents one fish (control: n = 99 fish; stressed: n = 94 fish). Data are represented as mean ± SEM.

To determine the effect of CUS on zebrafish behavior, we adapted assays classically used to measure anxiety-like behaviors in vertebrates. These consisted of an open field and a dark-light preference test that quantify anxiety in animal models based on the thigmotaxis and phototaxis behaviors respectively^15^. In the open field test, the fish trajectory was recorded as they swam in a round petri dish (Fig. 2A). We calculated a thigmotaxis index, reflecting the relative time spent along the walls of the arena, which is a common measure of anxiety-like behavior^33^. There was no difference in thigmotaxis between CUS-exposed and control fish (Fig. 2B). Basal locomotion, assessed via velocity and total distance swam, was similar across groups (Fig. 2C,D).

**Figure 3:**
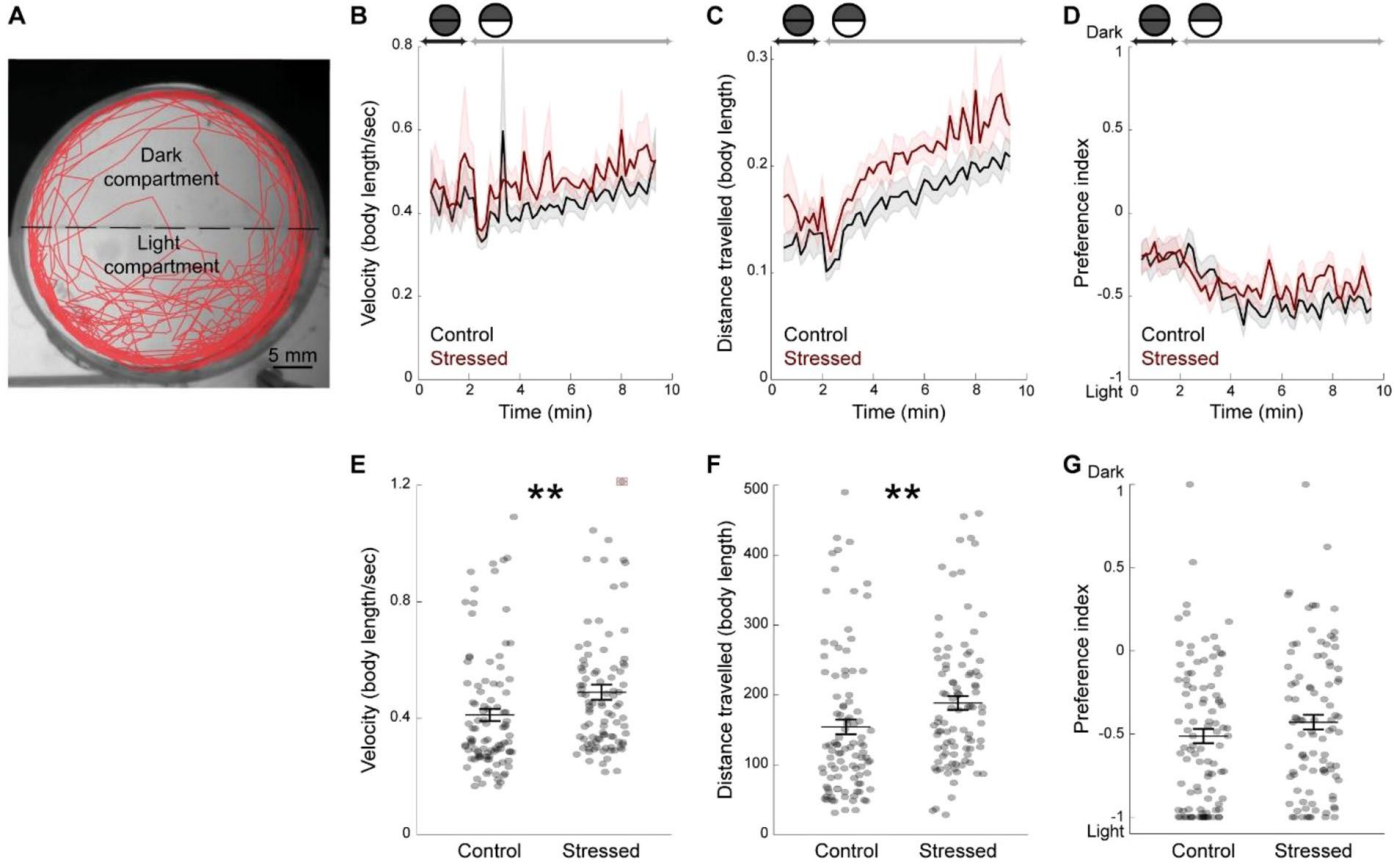
No change in light preference, but increased light-induced locomotion two days post-CUS, in CUS-exposed fish. (**A**) Representative swimming trajectory (in red) of a 19 dpf fish during the dark-light test. Fish were initially maintained in the dark for two minutes (dark phase). Upon the start of illumination (light phase), they had the choice between a dark and a light compartment for eight minutes. (**B**) Average velocity during the dark and light phases, per time bins of twelve seconds, in body length/second. (**C**) Average distance travelled during the dark and light phases, per time bins of twelve seconds, in body length. (**D**) Preference index over time, during the dark and light phases, calculated per time bins of twelve seconds. (**E**) Average velocity of the fish during the light phase. The dot signaled by a red square was out of range (value = 2.07). (**F**) Total distance travelled by the fish during the light phase. (**G**) Preference index for the dark or the light compartments during the light phase. Values of −1 indicate 100% time spent in the light compartment and values of 1 indicate 100% time spent in the dark compartment. Each dot represents one fish (control: n = 99 fish; stressed: n = 94 fish). Data are represented as mean ± SEM. **p < 0.01, Kruskal-Wallis test.

We next used a combination of two assays; the dark-light transition, which measures the change in locomotion resulting from sudden illumination, and the dark-light preference test, which measures the fish preference for the light over the dark compartment (Fig. 3A, see Methods). There was no difference in the number of freezing events (control: 13.1 ± 1.2; stressed: 9.8 ± 0.1; p value = 0.08; Kruskal-Wallis test) or in the duration of freezing events (in minute, control: 1.9 ± 0.2; stressed: 1.3 ± 0.2; p value = 0.06; Kruskal-Wallis test) between stressed and control groups. In both groups, fish exhibited a biphasic response following the transition from dark to light phase, which consisted in a short-term decrease in locomotion immediately after light onset (Fig. 3B), followed by an increase in distance swam in both groups (Fig. 3C). Interestingly, this light-induced locomotor response was more prominent in CUS-exposed fish than in control fish (Fig. 3E,F). We calculated the locomotion parameters in body length in order to account for the difference in size between stressed and control fish at 19 dpf (Fig. 1A). Measuring locomotion in mm showed a similar increase in light-induced response in CUS-exposed fish compared with control fish (velocity (mm/s); control: 1.5 ± 0.1; stressed: 1.7 ± 0.1; p value = 0.03; Kruskal-Wallis test; distance (mm), control: 651.5 ± 46.5; stressed: 728.6 ± 42.8; p value = 0.03; Kruskal-Wallis test). We also compared dark-light preference during the light phase of the assay. Fish avoided the dark compartment, as reflected by the negative average preference index that was similar in both groups (Fig. 3D,G).

### CUS exposure reduces exploration of a novel tank

**Figure 4:**
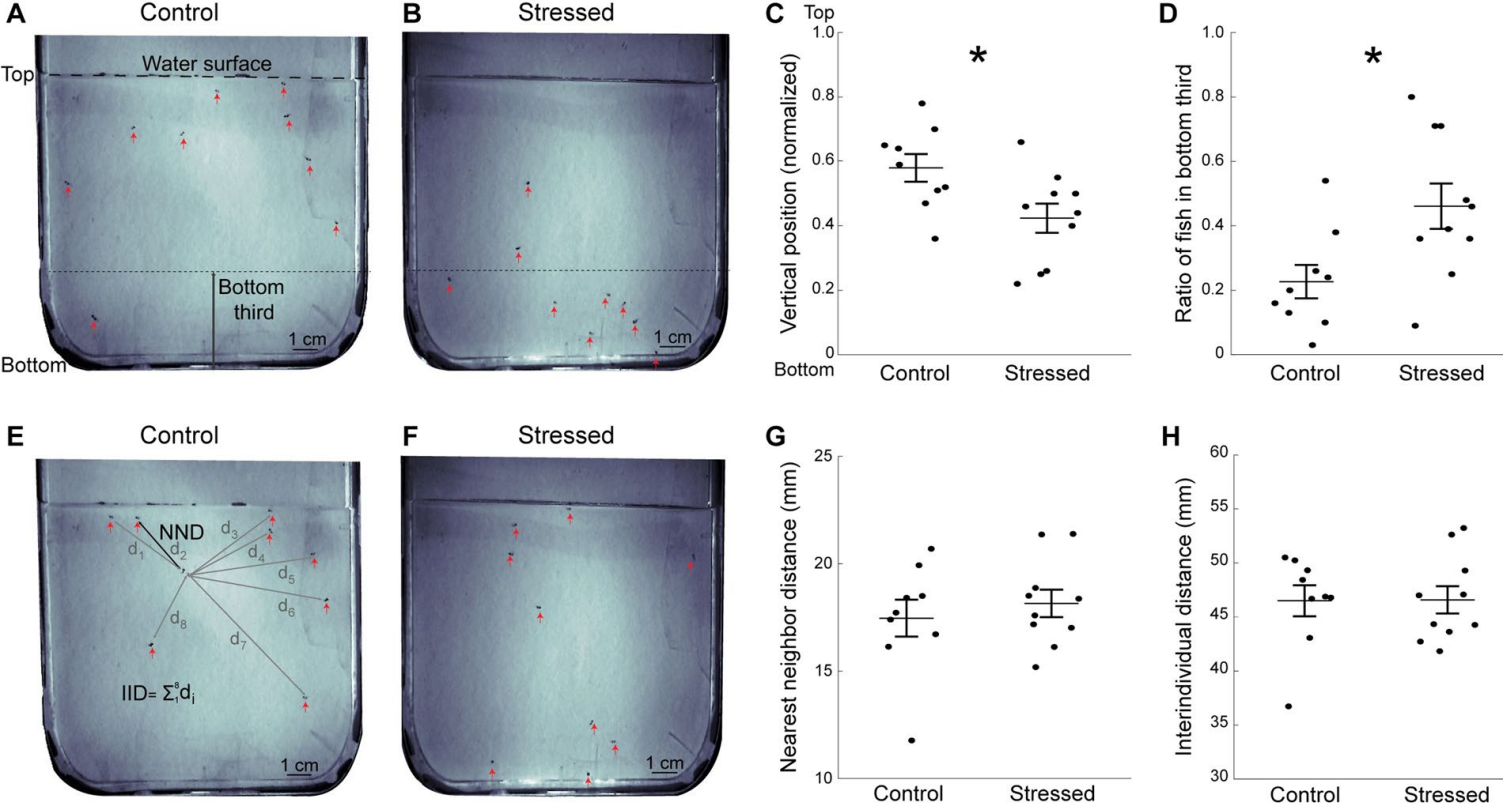
Decreased exploration of the novel tank in stressed fish tested one day post-CUS. (**A**) Representative image showing control fish’s position in the arena at the beginning of the novel tank test. The arena is vertically positioned and the dashed line at the top indicates the water surface. The red arrows indicate the fish, and the the bottom third of the tank is indicated. (**B**) Representative image showing stressed fish’s position at the beginning of the novel tank test. (**C**) Average vertical position of all fish during the early phase of the novel tank test (see Methods). (**D**) Ratio of fish in the bottom third of the tank. (**E**) Representative image showing control fish’s position relative to each other in the late phase (from three to ten minutes) of the novel tank test, in the same group as in A. The black line indicates the distance from a fish to its nearest neighbor (NND). The grey lines indicate the distance between one fish to the other fish in the tank that are used for interindividual distance (IID) calculations. (**F**) Representative image showing stressed fish’s position in the late phase of the novel tank test, in the same group as in (**B**). (**G**) Average nearest neighbor distance for all fish in the arena from three to ten minutes. (**H**) Average interindividual distance for all fish in the arena from three to ten minutes. Each dot represents the average value for one group of fish (control: n = 9 groups; stressed: n = 10 groups). Data are represented as mean ± SEM. *p < 0.05, Student’s *t*-test.

To further characterize stress-induced anxiety-like behaviors, groups of fish were introduced to a novel tank (Fig. 4A,B). There was no difference in the number of freezing events (control: 0.6 ± 0.1; stressed: 0.6 ± 0.14; p value = 1; Kruskal-Wallis test) or in the duration of freezing events (in second; control: 11.9 ± 4.9; stressed: 14.3 ± 3.7; p value = 0.27; Kruskal-Wallis test) between stressed and control groups. We also measured their exploration of this new environment, a parameter that is consistently reduced in anxious fish^23,34,35^. Chronic stressor exposure resulted in a significantly lower vertical position in the tank (Fig. 4C) and a higher proportion of fish swimming at the bottom of the tank (Fig. 4D).

**Figure 5:**
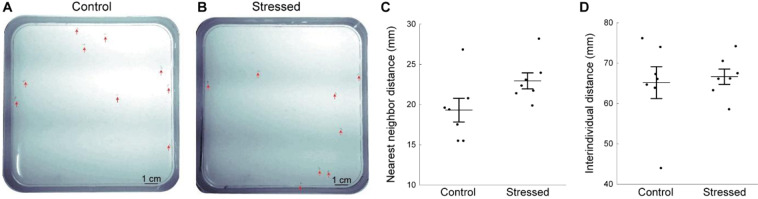
CUS exposure does not affect shoaling. Shoaling was measured for twenty minutes in a horizontal arena. (**A**) Representative image of the position of control fish relative to each other. (**B**) Representative image of the position of stressed fish relative to each other. Red arrows indicate the fish’s positions. (**C**) Average nearest neighbor distance for all fish in the arena. (**D**) Average interindividual distance for all fish in the arena. Each dot represents the average value for a group of fish (control: n = 7 groups; stressed: n = 7 groups). Data are represented as mean ± SEM.

Since zebrafish tend to swim in tighter shoals when exposed to direct or perceived threats^36^, we quantified shoaling by measuring the average distance between individuals as well as nearest-neighbor distance during the late part of the novel tank test (Fig. 4E,F). We found no difference in either of these parameters between the stressed and control fish (Fig. 4G,H). Since displacements constrained to the vertical dimension could affect social behavior, we also assessed shoaling in a horizontal arena, whose dimensions matched those used for larval fish in previous studies^37,38^ (Fig. 5A,B**)**. There was no difference in distance to nearest-neighbor (Fig. 5C), or in average distance between fish (Fig. 5D) between the stressed and control groups.

**Figure 6:**
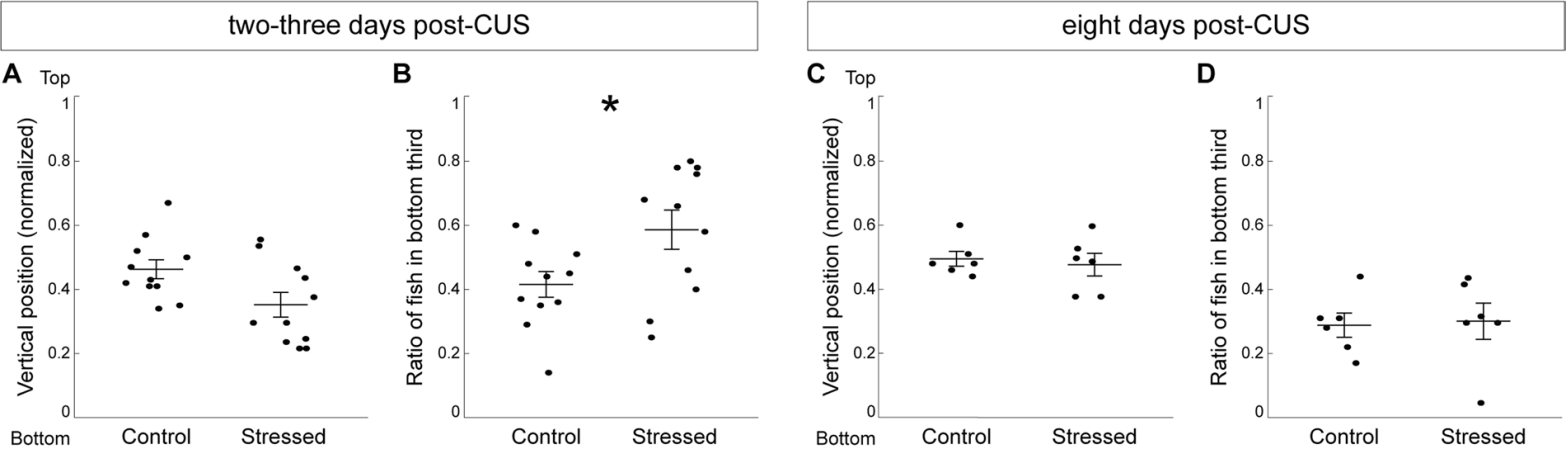
Decreased exploration of the novel tank persists three days post-CUS and returns to control level within a week. (**A**) Average vertical position in the novel tank test at two-three days post-CUS. (**B**) Ratio of fish in the bottom third at two-three days post-CUS (control: n = 11 groups; stressed: n = 11 groups). (**C**) Average vertical position in the novel tank test eight days post-CUS. (**D**) Ratio of fish in the bottom third eight days post-CUS. Each dot represents the average value for a group of fish (Control: n = 6 groups; Stressed: n = 6 groups). Data are represented as mean ± SEM. *p < 0.05, Student’s *t*-test.

Altogether, our results indicate that repeated stressor exposure for eight consecutive days induced an anxiety-like phenotype, which consists of heightened responsiveness to dark-light transition and decreased exploration in the novel tank test. To determine the duration of the anxious phenotype in CUS-exposed fish, we assessed their exploratory behavior two or three days, as well as eight days, post-CUS. We found that decreased exploration persisted in stressed fish at least three days after the last stressor was applied, as shown by the significantly higher proportion of stressed fish swimming at the bottom (Fig. 6B) and a tendency to swim lower in the tank (Fig. 6A). After one week, the anxious phenotype was no longer observed, as the exploration levels were similar in stressed and control fish (Fig. 6C,D).

## Discussion

The neurobiological consequences of prolonged stress have long been studied using chronic unpredictable stress (CUS) exposure in rodents^39–41^ and recently in adult zebrafish^14,22,30,42^. Adding to this, we present an adapted CUS protocol to investigate how chronic stress affects zebrafish during early-life stages. We used psychological and physiological aversive stimuli previously documented to activate the zebrafish stress response^22,24^. We found that repeated stressor exposure from 10 to 17 dpf significantly altered young zebrafish’s growth, as well as some aspects of anxiety-like behaviors.

Zebrafish larvae exposed to eight days of CUS were significantly smaller than their unstressed siblings. This observation was consistent with the decreased growth rates and reduced weight gain, reported in a variety of vertebrates during or shortly after prolonged stress exposure^31,42,43^. Fish in the control and stressed groups were fed equal amounts of larval food several times a day, thus the difference in growth cannot be explained by variation in food availability. However, it is well documented that reduced food intake is a common consequence of many types of stress and is partly mediated by the HPA axis^44–46^. Numerous studies have reported decreased appetite in adult fish following chronic stress^47,48^, and food intake was temporarily inhibited after exposure to strong stressors in developing zebrafish^25^. Therefore, the cumulative stress-mediated anorexia periods could account for the smaller size of CUS fish. Besides, cortisol-mediated proteolysis can directly affect muscle mass^32^, providing an additional mechanism through which CUS could have hampered fish growth. The decreased growth in stressed fish was temporary; stressed fish were the same size as age-matched unstressed siblings one week after the stressor had ceased. This is consistent with a previous report of body mass recovery after CUS exposure in developing salmon^31^ and has been attributed to post-stress compensatory feeding as observed in several fish species^31,49,50^.

Stressed fish showed reduced exploration of a novel environment in our study, which is in line with many other reports of acutely^23,51^ and chronically^22,30,42^ stressed fish. The novel tank test is a robust and widely used assay characterizing adult fish anxiety phenotypes^35,52^, that exploits the fish’s natural diving response when placed in a new environment. Despite the extensive use of this assay, little is known regarding the ontogeny of the zebrafish diving response. A recent study demonstrating larval preference for deeper waters suggests that this behavior could already emerge during the first week of life^53^. Here, we observed a robust diving response in groups of 18–20 dpf larvae, which lasted around two minutes (data not shown) and was consistently heightened by prior stress (Figs. 4C,D, 6B). Therefore, we show that the novel tank test is a suitable assay for measuring stress-induced anxious states in young zebrafish.

In contrast to prior studies using a single stressor exposure^28^, we found no difference in basal locomotion between CUS and control fish (Fig. 2C,D). However, stressed fish swam faster and over greater distances than their unstressed siblings after sudden illumination of the arena in the dark-light transition assay (Fig. 3B,C,E,F). Zebrafish larvae aged under one week react to sudden increased illumination by reducing locomotion^54^. We observed the opposite response pattern in three-weeks old fish, which exhibited a light-induced increase in locomotor activity. Since few prior studies have investigated the response to dark-light transitions in three-weeks old zebrafish, one needs to be cautious in interpreting this increased reactivity in terms of anxiety-related behavior. Interestingly, this effect is reminiscent of the enhanced behavioral reactivity induced by acute stress in response to changes in relevant environmental stimuli such as water temperature or vibrations^13^. Therefore, our data suggest that CUS specifically exacerbates the locomotor response to dark-light transitions, potentially reflecting an adaptive response to optimize coping with aversive environment changes.

Time spent close to the walls and dark-light preference are commonly used to assess fish anxiety-like behaviors at larval and adult stages^15,55^. Shoaling has also been used as an indicator of fear or anxiety states since adult zebrafish tend to group into tighter shoals when exposed to anxiogenic situations^22,56^. Although we observed significant effects of CUS on size, stimulus responsiveness and exploration of a novel environment, we found no effect on thigmotaxis, dark-light preference and shoaling. This could reflect the lower sensitivity of these assays at that developmental stage. Indeed, the three weeks-old zebrafish lie in a key dark-light preference transitory phase between phototactic larvae and negative phototactic adults^57^. In accordance with this, we found that 19 dpf zebrafish showed on average a moderate preference for the light compartment. However, the interindividual variability was very prominent (Fig. 3G), which could have masked subtle differences between the CUS and control fish. Regarding shoaling, fish were separated on average by 10 body lengths in the vertical novel tank test (Fig. 4H) and by 15 body lengths in an horizontal arena (Fig. 5D). This is similar to values reported for that developmental stage, but larger than the 4 body lengths between older shoaling fish^38^. Although preference for conspecifics is already strong in three-weeks old zebrafish^21,58,59^, our data confirm that shoaling, and by extension its modulation by stress^22^, develops at later stages. All together, our results underscore the importance of using age-appropriate and diverse behavioral assays. Moreover, it adds to the growing evidence documenting the differences in sensitivity and aspects of the anxiety-like response detected by different approaches^53,60^.

The behavioral outcomes of chronic stress exposure are most often assessed within 24 h after the last stressor^22,29,30^. We found that stressed fish displayed a decreased exploration of the novel tank for at least three days post-CUS and returned to normal levels after a week. The relatively short duration of the anxious phenotype must be placed in perspective with the mild stress protocol used here, to avoid deleterious effects on fish survival during the sensitive developmental period. Moreover, since we tested fish in groups in the novel tank test, the anxiolytic presence of conspecifics^61^ might have alleviated subtle anxiety phenotypes during the late post-CUS assessments. Early life stress can produce delayed effects on fish behaviors later in life^62–64^. Therefore, it would be interesting to compare the individual diving response and shoaling behavior of CUS and control fish during adulthood to determine the long-term persistence of the anxious phenotype.

## Methods

### Animals and housing

We used 548 zebrafish of following lines: nacre (mitfa)^65^, and Tg (elavl3: GCaMP6s)^66^. Eggs were obtained from crosses of adult fish raised in the facility. Eggs were collected between 10–12 am and kept until hatching at a density of 50 eggs/50 mL petridishes filled with artificial fish water. Fish were then transferred to new water tanks and raised at a constant temperature of 28 °C, under a 12/12 h light cycle and fed twice a day with commercial flakes (Tetra). All experiments were performed on zebrafish aged under 25 days post-fertilization (dpf) and the procedures followed the 2010/63/EU directive and were approved by the Norwegian Animal Research Authority (Food and Safety Authority; Permit number: 17127).

### Experimental design

**Figure 7:**
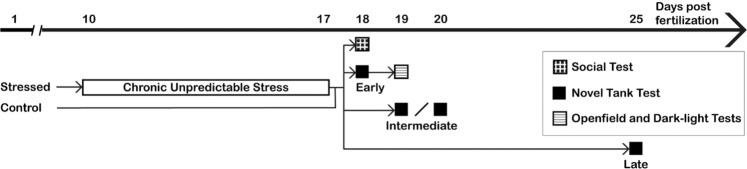
Experimental design. Fish in the stressed group were exposed to stressors for eight days. Fish in the control group were raised in the same conditions without stressor exposure. Social interaction was assessed in groups of fish at 18dpf (control: n = 59; stressed: n = 58). Early assessment of anxiety-like behaviors was done using the novel tank test at 18 dpf (control: n = 81; stressed: n = 83). The same fish were then tested in the open field and dark-light tests at 19 dpf (control: n = 99; stressed: n = 94). A separate group of fish was then tested in a novel tank for intermediate (19 or 20 dpf, (control: n = 95; stressed: n = 92)) or late (25dpf, (control: n = 24; stressed: n = 27)) assessment of anxiety-like behaviors.

Fish in the stressed group were exposed to chronic unpredictable stress (CUS) from 10 to 17 dpf (see Fig. 7). At that age, zebrafish possess a functional stress axis in response to external stressors^12,67^. Moreover, they are sufficiently small and transparent to permit *in vivo* functional brain imaging^68,69^ to investigate the effects of early life stress on neural activity in future studies. Control groups were raised in the same conditions without stressor exposure. Fish from both control and stressed groups were tested for social behaviour at 18 dpf. Another set of fish were tested for anxiety-like behaviors in the novel tank diving test (at 18 dpf; 81 control fish; 83 stressed fish), followed by the open field and dark-light tests (at 19 dpf; 99 control fish; 94 stressed fish). Note that all fish tested at 19 dpf had performed the novel tank test at 18 dpf. However, the video files for three groups (two control groups, n = 18 fish; and one stressed group, n = 11 fish) were overwritten, which prevented the inclusion of novel tank test results for these fish and explains the difference in animal numbers between 18 and 19 dpf. Additionally, fish that were left undisturbed after the end of the CUS were tested in the novel tank test at 19, 20 or 25 dpf to assess the persistence of the anxiety-like phenotype.

### Chronic unpredictable stress

At 10 dpf, fish were randomly assigned to control or stressed groups and housed in nursery tank with a meshed bottom that enabled easier transfer to the holding tank where CUS was applied. From 10 to 17 dpf, the fish in the stressed group were exposed to two stressors per day, applied at random times between 8 am and 8 pm to maintain unpredictability. The five following stressors were used according to previously published work^24,55^: (1) chasing (using a small net or a pipette for five minutes); (2) turbulences (tank water replacements followed by increased air bubbling for three minutes, repeated three times); (3) hyperosmotic shock (100 mM NaCl for ten minutes); (4) pH drop (pH = 4 for three minutes); (5) light flashes exposure (6 mW/cm^2^ light flashes at 5 Hz for ten minutes). The light flashes were produced by a custom-made panel of white LEDs controlled by a microcontroller board (Arduino Uno). Fish in the control group remained undisturbed except for routine feeding and tank cleaning.

### Behavioral assays

All recording sessions took place in the afternoon from 12–6 pm in a temperature-controlled room (28 °C) with limited experimenter intervention.

#### Novel tank diving test

Fish in groups of 5–11 (early and intermediate assessment groups) or 3–5 (late assessment group) were kept for one hour in temporary holding tanks, before being transferred to the novel arena (length × width × height: 13 ×1.4 × 6.4 cm). The fish movement was then recorded for ten minutes at 1 Hz using a webcam (Logitech 720p) placed in front of the arena and connected to a computer. The fish position was manually detected using the ImageJ ROI manager plugin^70^ (every five seconds for the first two minutes then every twenty seconds for the next eight minutes) and exported in Matlab (MathWorks 2017) to calculate the behavioral response indices. Vertical position for a group of fish was calculated by averaging all fish’s normalized vertical positions (bottom = 0 and water surface = 1). The first half a minute was not included in the analysis to avoid variations due to fish delivery. The fish vertical position and the ratio of fish in the bottom third were calculated for the following one minute. The ratio of fish in the bottom third is the average proportion of fish present in the bottom third of the tank during the specified period. The nearest-neighbor distance (NND) was calculated by averaging the distance to the closest neighbor for each fish within the group after the initial diving response took place (minutes three to ten). The interindividual distance (IID) was calculated as the average of all pairwise distances between individual fish within the group during the same period. Freezing episodes were defined as periods of immobility lasting for more than three seconds and were measured manually for the first three minutes of the assay by an experimenter blind to the group identity. The total number of freezing events and cumulative duration of freezing events were normalized to the number of fish within a group.

#### Shoaling test

Fish were transferred to the behavior testing room at least one hour prior to the experiments. Fish (6–10 per group) were gently transferred from the home tank to a centrifuge tube (Falcon, 50 ml) containing 15 ml of fish water and were poured into the center of a square petri dish (length × width × height: 12 × 12 × 1.5 cm) containing 60 mL of water. The petri dish arena had linear dimensions over 28 times the average fish body length, as recommended in previous studies of shoaling behavior^37,38^. The fish movement was then recorded for 20 minutes at 1 Hz using a webcam (Logitech 720p) placed above the arena and connected to a computer. The individual fish position was manually detected using the ImageJ ROI manager plugin (every 10 seconds during minutes 3, 5, 7, 9, 15 and 20) and exported in Matlab to calculate NND and IID as described above.

#### Open field and dark-light tests

Fish were transferred in individual petridishes (radius = 2 cm, depth = 0.4 cm; Falcon) and habituated for 1 h. The dishes were then positioned on a transparent platform illuminated from below using visible and infrared LEDs. The walls of the dishes were sanded to prevent visual and social interaction between fish. A camera (AVT Manta G-235B; Allied Vision) equipped with an infrared high-pass filter (cut-off wavelength 750 nm; ePlastics) was positioned above the platform and used to simultaneously record the behavior of sixteen fish at 2 Hz. An enclosure positioned around the setup prevented visual interference. A recording of 10 minutes was taken for the open field test. Then the visible-light LEDs were switched off and the walls and bottom of halves of each petridishes were covered with black infrared-transparent material (cut-off wavelength 750 nm; ePlastics). After five minutes of habituation to the new settings in the dark, the fish position in the dark-light partitioned dish was recorded for ten minutes: two minutes in the dark (dark phase) followed by eight minutes with visible light illumination (light phase). This allowed us to first test the response to change in background illumination (dark-light transition) and then the fish preference for the dark or the light compartment (dark-light preference).

To reconstruct the fish swimming trajectory, the video recordings were analyzed using custom-written Matlab scripts^71^. The following behavior indices were then calculated based on fish position. Freezing episodes were defined as periods of immobility lasting more than three seconds, during which the fish’s speed was inferior to a fifth of its body length. Velocity was quantified in body length/sec after excluding freezing episodes. Distance travelled corresponded to the cumulative displacement of the fish. For the calculation of the open field thigmotaxis index, the petridish was divided into three equal-area concentric circles. The relative time spent by fish in the outermost circle during the test was then calculated, with a value close to 100% indicating a strong preference for the border of the dish, whereas values close to 0% indicated a preference for the center. The preference index in the dark-light test was calculated as follows^57^:

$$preference\,index=\frac{(time\,in\,dark-time\,in\,light)}{total\,duration}$$

Values close to −1 indicated a strong preference for the light compartment; values close to 1 indicated a strong preference for the dark compartment and values close to 0 indicated no preference.

### Measurement of fish size

At 19 dpf, the fish body length was calculated in Matlab for each fish by averaging snout-to-tail measurements on five frames randomly selected from the open field test recordings. At 25 dpf, fish were euthanized using an overdose of 222 mg/L buffered tricaine methane sulfonate (MS222). They were placed laterally on a petridish and snout-to-tail measurements were made on pictures taken using a microscope camera (Axiocam 506, ZEISS).

### Statistical analysis

Data were evaluated using SPSS Statistics 25 (IBM). Each data set was first tested for normality with descriptive statistics for mean, skewness and kurtosis. Group effects were then tested using Student’s t-test or non-parametric Kruskal-Wallis test. Data are reported as mean ± standard error of the mean (SEM) in the text and figure legends. A p-value < 0.05 was considered significant.
